# Diagnostic utility of magnetic resonance urography in pediatric ureteropelvic junction obstruction: evaluating changes in anteroposterior diameter of the renal pelvis following furosemide administration

**DOI:** 10.1007/s00247-025-06393-1

**Published:** 2025-11-10

**Authors:** Charlotte Grognet, Héloïse Lerisson, Daniela Rapilat, Nathalie Boutry, René-Hilaire Priso, Dyuti Sharma, Arthur Lauriot Dit Prevost, Philippe Puech

**Affiliations:** 1 Department of Pediatric Imaging, Centre Hospitalier Universitaire de Lille, Hôpital Jeanne de Flandre, Avenue Eugène Avinée, 59000 Lille, France; 2 Department of Pediatric Surgery, Centre Hospitalier Universitaire de Lille, Hôpital Jeanne de Flandre, Avenue Eugène Avinée, 59000 Lille, France; 3 Department of Genitourinary and Women’s Imaging, Centre Hospitalier Universitaire de Lille, Hôpital Claude Huriez, Rue Michel Polonowski, 59000 Lille, France

**Keywords:** Child, Magnetic resonance urography, Renal pelvis, Ureteropelvic junction obstruction

## Abstract

**Background:**

Magnetic resonance urography (MRU) with diuretic injection is increasingly used to evaluate pediatric ureteropelvic junction obstruction, providing anatomical and functional information without ionizing radiation.

**Objective:**

To analyze the increase in anteroposterior diameter of the renal pelvis following furosemide injection during MRU to determine a diagnostic cutoff. To assess whether this increase supports etiological diagnosis and whether magnetic resonance imaging (MRI) reliably detects crossing vessels.

**Materials and methods:**

We retrospectively included all children who underwent surgery for ureteropelvic junction obstruction at our institution between January 2010 and January 2023 and had preoperative MRU. For each patient, the increase in renal pelvis diameter after furosemide injection during MRU was measured on the obstructed and contralateral healthy sides. Measurements were compared to determine a pathological cutoff. The change in renal pelvis diameter was also compared according to etiology (intrinsic vs. extrinsic). The association between crossing vessels identified on MRI and intraoperative findings was also assessed.

**Results:**

Seventy patients (median age 9) were included. The increase in renal pelvis diameter was significantly greater on the obstructed side compared to the contralateral side (*P* < 0.001). The optimal cutoff for predicting obstruction was 6 mm (sensitivity 68.6%, specificity 87.1%). No significant difference was found in the change in renal pelvis diameter according to etiology (*P* = 0.86). The association between crossing vessels identified on MRI and during surgery was significant (*P* < 0.001).

**Conclusion:**

An increase greater than 6 mm in renal pelvis diameter after furosemide injection during MRU could represent an additional diagnostic criterion for pediatric ureteropelvic junction obstruction.

**Graphical Abstract:**

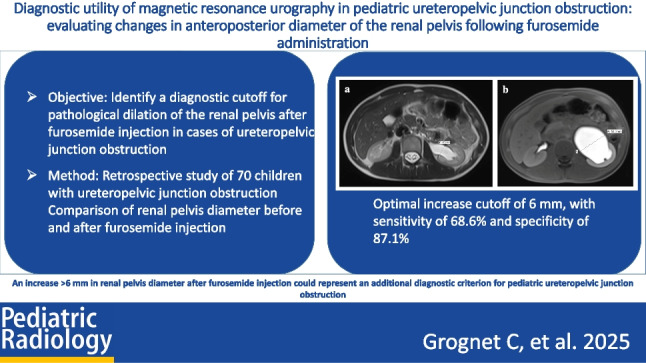

## Introduction

Ureteropelvic junction obstruction is defined as an obstruction of urine flow from the renal pelvis into the proximal ureter, resulting in pelvicalyceal dilation [1]. It is the most common cause of hydronephrosis in pediatric patients, with an estimated incidence of 1 in 1,500 live births [1]. Two main types of etiology are classically distinguished [2]. Intrinsic obstructions include congenital stenoses and peristaltic defects of the ureteropelvic junction, and are more common in neonates and infants [3]. Extrinsic obstructions are mainly due to compression of the ureteropelvic junction by a crossing lower pole renal vessel and are more common in children and adolescents [4].

Ureteropelvic junction obstruction may be suspected antenatally through the detection of fetal hydronephrosis, or later in childhood in cases of lower back pain, sometimes associated with nausea or vomiting, or during investigation of recurrent urinary tract infections [4, 5]. Chronic obstruction can lead to recurrent lower back pain, lithiasis, urinary tract infections and impaired renal function [3].

Approximately one-third of patients require surgical management [4]. The reference surgical treatment is Anderson-Hynes pyeloplasty, with a success rate of about 95% [4]. Surgical planning for ureteropelvic junction obstruction correction takes into account the possibility of a crossing vessel, highlighting the importance of preoperative imaging.

Ureteropelvic junction obstruction requires additional morphological and functional investigations to provide the information necessary for management. Ultrasound is the imaging modality of choice for diagnosing hydronephrosis, but it cannot confirm obstruction or assess its functional impact [6]. Dynamic renal scintigraphy can quantify differential renal function and confirm obstruction in ureteropelvic junction obstruction, but it involves exposure to ionizing radiation [3]. Magnetic resonance urography (MRU) is increasingly used, as it provides both anatomical and functional information without ionizing radiation [7]. It can confirm obstruction at the ureteropelvic junction, identify potential causes such as a crossing vessel, and assess its severity [8]. The administration of a diuretic (furosemide) during the examination aims to improve diagnostic sensitivity for ureteropelvic junction obstruction by increasing pelvicalyceal dilation [9]. This may induce lower back pain during the examination, which is itself a sign of an obstruction. Magnetic resonance urography also allows for a functional assessment to confirm the obstruction and evaluate differential renal function [7].

Diagnosing equivocal forms of ureteropelvic junction obstruction, characterized by minimal pelvicalyceal dilation or non-specific pain, can be challenging. Several studies have investigated the increase in renal pelvis diameter during dynamic ultrasound following diuretic administration for the assessment of ureteropelvic junction obstruction [10, 11]. These studies have shown that this approach may help confirm upper urinary tract obstruction. However, under normal hydration conditions, furosemide-induced diuresis can lead to renal pelvis enlargement even in the absence of anatomical or functional obstruction. To our knowledge, no study has assessed the increase in renal pelvis diameter after diuretic administration during MRU in pediatric ureteropelvic junction obstruction by comparing obstructive and non-obstructive renal pelvises. This approach could help to confirm obstruction in ureteropelvic junction obstruction and provide an additional diagnostic criterion. Moreover, identifying a crossing vessel as the cause of ureteropelvic junction obstruction remains difficult on magnetic resonance imaging (MRI), despite its importance in surgical planning. It is therefore necessary to identify predictive factors for the etiological role of a crossing vessel.

The aim of this study is to analyze the increase in the anteroposterior diameter of the renal pelvis between sequences performed before and after furosemide injection during MRU in pediatric ureteropelvic junction obstruction, in comparison with the contralateral normal pelvis, to determine a pathological cutoff for the positive diagnosis. The study also aims to evaluate whether this increase can support the etiological diagnosis of ureteropelvic junction obstruction and to assess the association between the detection of a crossing vessel on preoperative MRI and intraoperative findings.

## Materials and methods

### Patients

This retrospective study was conducted in the pediatric imaging department of  Lille University Hospital. All patients who underwent pyeloplasty for ureteropelvic junction obstruction in the pediatric surgery department between January 2010 and January 2023 and had a preoperative MRU were included. At the study center, MRI was performed as a second-line investigation following ultrasound in children and adolescents, and in selected cases in younger children. Patients with a history of a single kidney, upper urinary tract malformations, bilateral ureteropelvic junction obstruction, previous upper urinary tract surgery, or renal transplantation were excluded. Patients were also excluded if the operative report was unavailable in the electronic medical record, if the MRU protocol was incomplete, or if the MRU sequences were severely affected by artifacts, preventing accurate measurement of renal pelvis diameter.

### Data

Clinical data including age, sex, medical history and operative reports were retrospectively collected from the electronic medical record system Sillage (Euris, Paris, France). We recorded any history of lower back pain or renal colic, urinary tract infection, and antenatal diagnosis of malformative uropathy. The surgical technique and the presence of a crossing vessel during surgery were retrieved by reviewing operative reports. The etiology of ureteropelvic junction obstruction (extrinsic compression by a crossing vessel or intrinsic cause) was determined based on the intraoperative identification or absence of a crossing vessel responsible for the obstruction.

Data from imaging and nuclear medicine tests were retrospectively collected from the picture archiving and communication system IntelliSpace PACS (Philips Healthcare, Best, Netherlands) used at the study center. Scintigraphy reports were analyzed for evidence of impaired differential renal function (< 40%). In our center, scintigraphy was mainly indicated as a second-line investigation after ultrasound in infants with marked or increasing hydronephrosis, and in older children as a complement to ultrasound and MRI when renal function remained uncertain or in asymptomatic patients to assess cortical transit time.

This retrospective study was conducted in compliance with institutional regulations and was declared to the national data protection authority. Patients and their legal guardians were informed that their anonymized medical data could be used for research purposes, and none objected. Formal IRB approval was not required for this type of study, in accordance with national legislation.

### Magnetic resonance urography

**Fig. 1:**
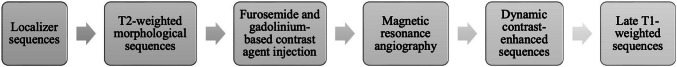
Magnetic resonance urography protocol

Examinations were performed on 1.5 Tesla MRI scanners: Magnetom Aera (Siemens Healthineers, Erlangen, Germany) or Ingenia (Philips Healthcare, Best, Netherlands). Magnetic resonance urography was scheduled in the morning without prior fasting. Patients were therefore presumed to be adequately hydrated at the time of the examination. The imaging protocol included localizer sequences, ultra-fast T2-weighted spin echo sequences in the three anatomical planes, and a coronal or 3D heavily T2-weighted sequence. Following intravenous administration of furosemide (Furosemide Kalceks, 20 mg/2 ml; Ever Pharma, Unterach am Attersee, Austria; 1 mg/kg, maximum 20 mg) and a gadolinium-based contrast agent—selected according to the child’s age (Dotarem [Guerbet, Villepinte, France], ProHance [Bracco Diagnostics, Monroe Township, NJ], Gadovist [Bayer Healthcare, Berlin, Germany], or Clariscan [GE Healthcare, Oslo, Norway])—the protocol proceeded with a magnetic resonance angiography sequence, coronal T1-weighted dynamic contrast-enhanced sequences, and delayed axial and coronal T1-weighted sequences with fat saturation (Fig. 1). General anesthesia was used in certain cases depending on the age of the patient and the level of agitation.

**Fig. 2:**
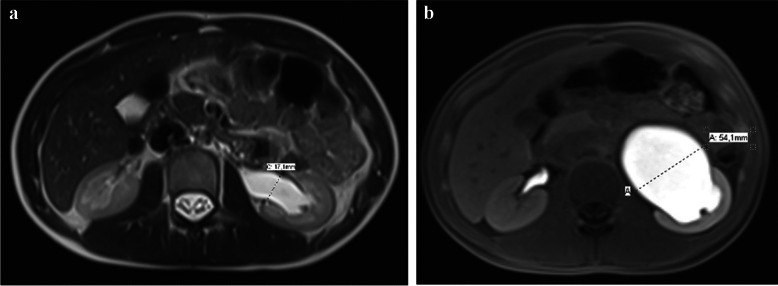
Measurements of the anteroposterior diameter of the renal pelvis before and after furosemide injection in a 14-year-old girl with left-sided ureteropelvic junction obstruction. **a** Axial T2-weighted MRI image before furosemide injection. **b** Axial fat-saturated T1-weighted MRI image after intravenous injection of furosemide and a gadolinium-based contrast agent* (images from the pediatric imaging department of Lille University Hospital)*

For each patient, measurements of the anteroposterior diameter of the renal pelvis were performed on both the hydronephrotic and contralateral normal side, using axial images. Measurements were taken either intra- or extra-sinusally depending on the location of the maximal diameter, both before and after the injection of furosemide and gadolinium (Fig. 2).

**Fig. 3:**
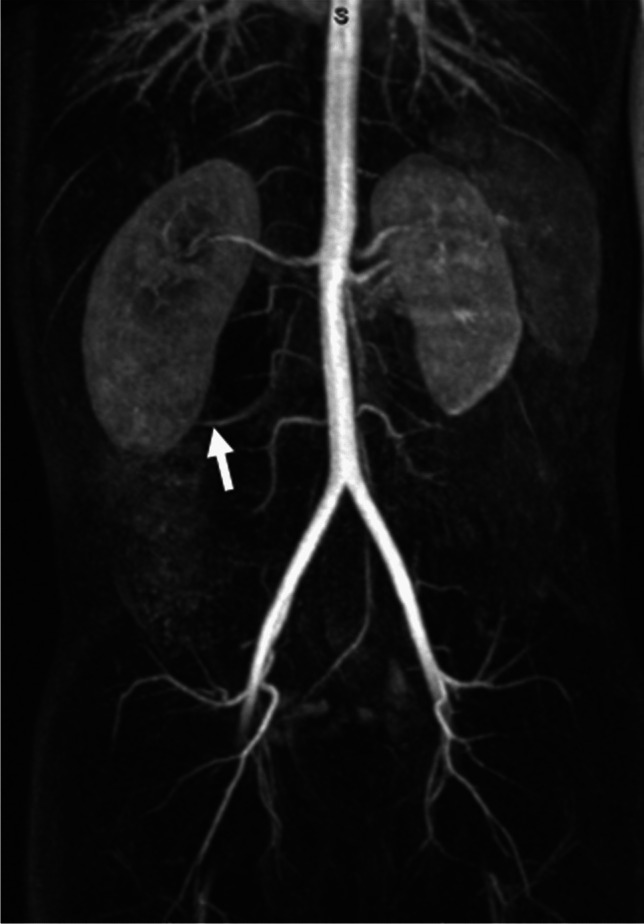
A 9-year-old boy with right-sided ureteropelvic junction obstruction. Coronal maximum intensity projection (MIP) reconstruction from a contrast-enhanced magnetic resonance angiography sequence shows a right lower pole crossing vessel (*arrow*) *(image from the pediatric imaging department of Lille University Hospital)*

The anteroposterior diameter after furosemide injection was measured on the final post-injection sequence. The side of ureteropelvic junction obstruction was recorded, and the presence of a crossing vessel was assessed on the magnetic resonance angiography sequence (Fig. 3). The time interval between furosemide administration and the acquisition of the post-injection sequence used for renal pelvis measurements was calculated. Bladder filling at the time of examination was assessed semi-quantitatively as low, partial, or full. Patients were asked to void prior to the scan.

All measurements were performed retrospectively by the same operator, a radiologist with 5 years of experience (C.G.)

### Statistical analysis

Qualitative variables were described using counts and percentages. Quantitative variables were described using the median and interquartile range (i.e. 25th and 75th percentiles). The normality of distributions was tested using the Shapiro–Wilk test and visually assessed with histograms.

The increase in renal pelvis diameter after furosemide injection was compared between the healthy and ureteropelvic junction obstruction affected sides within the same patient using a linear mixed-effects model, adjusted for baseline renal pelvis diameter before furosemide injection. This model allowed for analysis of covariance (ANCOVA) with repeated measures while accounting for intra-subject correlation. The choice of covariance structure was based on the Akaike Information Criterion (AIC).

The increase in renal pelvis diameter on the pathological side after furosemide injection was compared between intrinsic and extrinsic obstruction (i.e., crossing vessel) using an analysis of covariance (ANCOVA), adjusted for baseline renal pelvis diameter before furosemide injection on the affected side.

An optimal cutoff for the increase in renal pelvis diameter after furosemide injection to differentiate between obstructive and non-obstructive pelvises was determined by maximizing the Youden index, calculated from the marginal probabilities of a generalized linear mixed model. The performance of this cutoff was described in terms of sensitivity and specificity.

For each model, residual normality was assessed.

The association between the presence of a crossing vessel on MRI and intraoperative findings was evaluated using a Chi-squared test.

All analyses were performed using SAS software (version 9.4; SAS Institute Inc., Cary, NC). Statistical tests were two-sided, with a significance level set at 5%.

## Results

### Patients

**Fig. 4:**
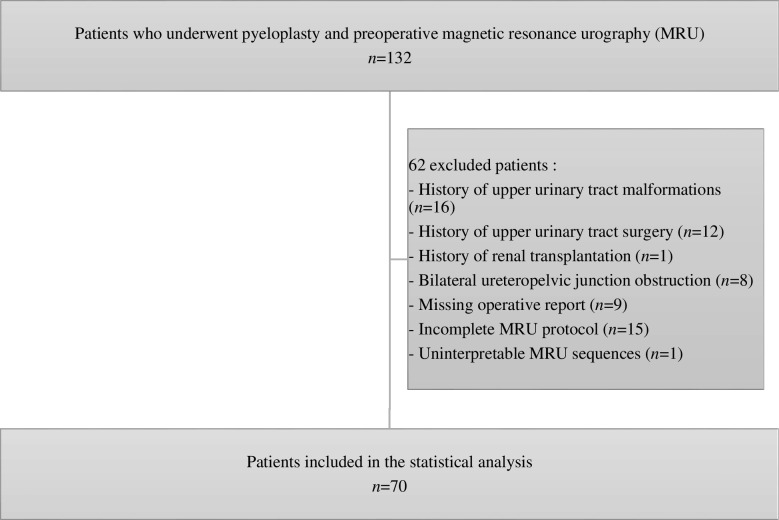
Flowchart of the study

A total of 132 patients with preoperative MRU underwent pyeloplasty in the pediatric surgery department of our institution between January 2010 and January 2023. After the exclusion of 62 patients, 70 were included in the statistical analysis (Fig. 4). Among those excluded for a history of upper urinary tract malformations, 1 patient had a single kidney, 10 had a horseshoe kidney, 4 had a duplex kidney, and 1 had a multicystic dysplasia of the right kidney associated with a pelvic left kidney. Of the 15 patients excluded due to an incomplete MRU protocol, 2 had not received furosemide injection. Among the 70 patients included in the statistical analysis, five underwent MRI under general anesthesia, aged 2, 3, 4, 5, and 6 years.

The characteristics of the patients are shown in Table 1. Of the 70 patients included, 42 were boys. Age ranged from 0 to 16 years, with a median of 9 years (interquartile range: 6–12 years). Twenty-one patients had a history of urinary tract infection, 50 had a history of lower back pain or renal colic, and 22 had a history of antenatal diagnosis of malformative uropathy. Ureteropelvic junction obstruction was left-sided in 69 patients and right-sided in 21. Fifty-five patients underwent preoperative renal scintigraphy (diethylenetriamine pentaacetic acid or mercaptoacetyltriglycine), which showed impaired differential renal function in 24 cases. Extrinsic compression by a crossing vessel was identified during surgery in 36 patients, while 34 had ureteropelvic junction obstruction of intrinsic etiology. Sixty-six patients underwent pyeloplasty using the Anderson-Hynes technique. This was combined with uncrossing of a lower pole renal vessel in 32 cases. One patient underwent transposition of a lower pole crossing vessel without pyeloplasty (vascular hitch) and 3 patients had other surgical techniques adapted according to intraoperative findings.

**Table 1:** Characteristics of the patients (qualitative variables are described as counts and percentages; quantitative variables are described as median and interquartile range)

Variables		Missing data	Extrinsic compression by a crossing vessel		Intrinsic cause		Total	
			*n* = 36		*n* = 34		*n* = 70	
Age *(years)*		0	9.5	(6–12)	9	(5–11)	9	(6–12)
Sex		0						
	Boy		19	(52.8%)	23	(67.7%)	42	(60.0%)
	Girl		17	(47.2%)	11	(32.3%)	28	(40.0%)
History of urinary tract infection		0						
	No		27	(75.0%)	22	(64.7%)	49	(70.0%)
	Yes		9	(25.0%)	12	(35.3%)	21	(30.0%)
History of lower back pain or renal colic		0						
	No		4	(11.1%)	16	(47.1%)	20	(28.6%)
	Yes		32	(88.9%)	18	(52.9%)	50	(71.4%)
Antenatal diagnosis		0						
	No		29	(80.6%)	19	(55.9%)	48	(68.6%)
	Yes		7	(19.4%)	15	(44.1%)	22	(31.4%)
Side of ureteropelvic junction obstruction		0						
	Right		10	(27.8%)	11	(32.4%)	21	(30.0%)
	Left		26	(72.2%)	23	(67.6%)	49	(70.0%)
DRF < 40%		15						
	No		15	(53.6%)	16	(59.3%)	31	(56.4%)
	Yes		13	(46.4%)	11	(40.7%)	24	(43.6%)
Crossing vessel on MRI		0						
	No		5	(13.9%)	25	(73.5%)	30	(42.9%)
	Yes		31	(86.1%)	9	(26.5%)	40	(57.1%)
Crossing vessel responsible of obstruction during surgery		0						
	No		0	(0%)	34	(100%	34	(48.6%)
	Yes		36	(100%)	0	(0%)	36	(51.4%)
Surgical technique		0						
	Anderson-Hynes pyeloplasty		0	(0%)	34	(100%)	34	(48.6%)
	Anderson-Hynes pyeloplasty with uncrossing of a lower pole renal vessel		32	(88.9%)	0	(0%)	32	(45.7%)
	Vascular hitch		1	(2.8%)	0	(0%)	1	(1.4%)
	Other		3	(8.3%)	0	(0%)	3	(4.3%)

*DRF* differential renal function, *MRI* magnetic resonance imaging

### Increase in renal pelvis diameter after furosemide injection during magnetic resonance urography

Table 2 summarizes the results of anteroposterior renal pelvis diameter measurements before and after furosemide injection on the healthy side and on the ureteropelvic junction obstruction side. The median renal pelvis diameter before furosemide injection was 24 mm (interquartile range: 18–34 mm) on the ureteropelvic junction obstruction side, compared with 6 mm (interquartile range: 3–8 mm) on the healthy side. Measurements of the maximal anteroposterior diameter after furosemide injection were taken between 7 and 175 min after diuretic administration. The median diameter of the renal pelvis after furosemide injection was 37 mm (interquartile range: 30–46 mm) on the ureteropelvic junction obstruction side, compared with 9 mm (interquartile range: 7–12 mm) on the healthy side. The median increase in renal pelvis diameter after furosemide injection was 8 mm (interquartile range: 5–13 mm) on the ureteropelvic junction obstruction side, compared with 4 mm (interquartile range: 3–5 mm) on the healthy side. The increase in renal pelvis diameter after furosemide injection was significantly greater on the ureteropelvic junction obstruction side compared with the healthy side (*P* < 0.001). The optimal cutoff for the increase in renal pelvis diameter after furosemide injection for predicting pathological obstruction was 6 mm. This cutoff was determined by maximizing the Youden index and provided the best diagnostic performance, with good sensitivity (68.6%) and very good specificity (87.1%).

**Table 2:** Measurements of the anteroposterior diameter of the renal pelvis before and after furosemide injection during magnetic resonance urography (qualitative variables are described as counts and percentages; quantitative variables are described as median and interquartile range)

Variables		Missing data	Extrinsic compression by a crossing vessel *n* = 36		Intrinsic cause *n* = 34		Total *n* = 70	
Diameter of the renal pelvis before furosemide injection on the affected side *(mm)*		0	26.5	(19–34)	23	(16–33)	24	(18–34)
Diameter of the renal pelvis after furosemide injection on the affected side *(mm)*		0	37.5	(31–48)	36.5	(29–43)	37	(30–46)
Increase in renal pelvis diameter after furosemide injection on the affected side *(mm)*		0	7.5	(5–15)	9	(6–13)	8	(5–13)
Diameter of the renal pelvis before furosemide injection on the healthy side *(mm)*		0	6	(3–7)	5.5	(3–8)	6	(3–8)
Diameter of the renal pelvis after furosemide injection on the healthy side *(mm)*		0	9	(7–12)	9	(7–12)	9	(7–12)
Increase in renal pelvis diameter after furosemide injection on the healthy side *(mm)*		0	4	(2–4)	4	(3–5)	4	(3–5)
Time between furosemide injection and sequence used for renal pelvis measurement *(minutes)*		0	25.4	(14–88)	23	(18–79)	23.8	(16–81)
Degree of bladder filling during MRU		0						
	Low		19	(52.8%)	19	(55.9%)	38	(54.3%)
	Partial		12	(33.3%)	12	(35.3%)	24	(34.3%)
	Full		5	(13.9%)	3	(8.8%)	8	(11.4%)

*MRU* magnetic resonance urography

We also compared the increase in renal pelvis diameter after furosemide injection on the ureteropelvic junction obstruction side according to etiology. The median increase in renal pelvis diameter after furosemide injection was 7.5 mm (interquartile range: 5–15 mm) in cases of extrinsic compression by a crossing vessel, compared with 9 mm (interquartile range: 6–13 mm) in cases of intrinsic obstruction. There was no significant difference in the increase in renal pelvis diameter after furosemide injection between ureteropelvic junction obstruction due to intrinsic causes and that due to extrinsic compression by a crossing vessel (*P* = 0.86).

### Presence of a crossing lower pole renal vessel on magnetic resonance imaging

A crossing vessel was identified on MRI in 40 patients. In 5 of these patients, it was not confirmed during surgery. In 4 patients, a crossing vessel was found during surgery but was not responsible for ureteropelvic junction obstruction. MRI failed to identify a crossing vessel responsible for ureteropelvic junction obstruction in 5 patients. The association between the presence of a crossing vessel on MRI and during surgery was statistically significant (*P* < 0.001). The diagnostic performance of MRI in detecting a crossing vessel is summarized in Table 3.

**Table 3:** Diagnostic performance of magnetic resonance imaging for the detection of a crossing vessel

Crossing vessel	Surgery +	Surgery -	Total	
**MRI + **	35	5	40	PPV = 87.5%
**MRI -**	5	25	30	NPV = 83.3%
**Total**	40	30	70	
	Se = 87.5%	Sp = 83.3%		

*MRI magnetic resonance imaging, NPV* negative predictive value, *PPV* positive predictive value, *Se* sensitivity, *Sp* specificity

## Discussion

The results of this study show that a significant increase in the renal pelvis diameter after furosemide injection, with a diagnostic cutoff of 6 mm, provides an additional argument for the positive diagnosis of ureteropelvic junction obstruction on MRU in children, with high sensitivity and specificity. Further studies are needed to confirm these findings. Measurements in this study were standardized on axial sections to maximize reproducibility. As the onset of action of intravenous furosemide occurs within a few minutes and its duration of action lasts 2 to 3 h, all post-injection measurements were performed under the effect of the diuretic [12]. However, we observed high variability in the time between furosemide injection and acquisition of the late sequences (7–175 min), mainly because imaging performed under general anesthesia did not allow for very late sequences. Standardizing this parameter in future studies could improve the consistency of results. The reliability of measurements is questionable in 8 patients in whom MRU was performed with a full bladder, despite the precautions of our protocol. Indeed, a full bladder is both a factor inhibiting emptying of the pelvicalyceal system and a source of variability in pelvicalyceal dilation, which could artificially increase renal pelvis measurements [3, 13]. Furthermore, there was no standardization of the patients’ hydration status before the examination and furosemide injection.

Since compression of the ureteropelvic junction by a crossing vessel is the most common cause of ureteropelvic junction obstruction in children and adolescents, the presence of such a vessel must be systematically sought to optimally guide surgical management [4]. Indeed, the presence of a crossing vessel may suggest a different surgical approach and could be a source of hemorrhagic complications or renal ischemia in the event of injury during surgery [14]. In this study, MRI showed very good agreement with intraoperative findings for detecting crossing vessels. These results are consistent with the existing literature [15, 16]. However, the presence of a crossing vessel on MRI does not necessarily imply its involvement in the obstruction. In fact, 4 patients in this study had a crossing vessel identified on MRI and found during surgery, but it was not responsible for the obstruction of the ureteropelvic junction. Identifying predictive factors for the etiological role of a crossing vessel in ureteropelvic junction obstruction appears necessary. This study showed no significant difference in the increase in renal pelvis diameter after furosemide injection between ureteropelvic junction obstruction due to intrinsic causes and that due to extrinsic compression by a crossing vessel. These results are in line with data from a study evaluating MRI in detecting crossing vessels and their clinical significance [15]. Other predictive factors were also assessed in that study, notably the presence of parenchymal edema on T2-weighted sequences, parenchymal thinning, or stasis in the proximal ureter, but none of these results was significant [15]. Therefore, MRI is the imaging modality of choice for detecting crossing vessels, but it cannot reliably determine whether they are the cause of obstruction in ureteropelvic junction obstruction.

This study has several limitations. First, the small number of patients included may limit the generalizability of the results. Larger studies, including multicenter studies, are therefore needed. Second, this was a retrospective study of patients who had undergone preoperative MRU. However, preoperative MRU is not routinely performed, particularly in infants and young children, in whom the most common cause of ureteropelvic junction obstruction is intrinsic and general anesthesia is more often required [3]. This category of patients was underrepresented in our study, although the patients’ ages ranged from 0 to 16 years. Patients with a history of upper urinary tract malformations were excluded, making it impossible to apply the results to this population. The same applies to patients with a history of upper urinary tract surgery, particularly those who had already undergone surgery for ureteropelvic junction obstruction, in whom a recurrence may be suspected.

MRI is a promising imaging modality for investigating ureteropelvic junction obstruction in children. It provides an accurate anatomical assessment of the entire urinary tract and a functional assessment, including confirmation of obstruction and quantification of differential renal function, all without exposure to ionizing radiation [8]. A prospective study showed that MRU provided information equivalent to scintigraphy regarding function, and superior information compared to ultrasound and scintigraphy regarding morphology [17]. Another study proposed a score based on MRU to assist with surgical indications for ureteropelvic junction obstruction in children [18]. This score included analysis of the excretory phase, presence of an abnormal vessel, and an anteroposterior diameter of the renal pelvis greater than 23 mm. This study also showed that patients who underwent surgery had greater pelvicalyceal dilation, which increased more after furosemide injection, compared with patients who received follow-up care. The availability of MRI, its cost, and sometimes the need for sedation or general anesthesia in young children limit its use and currently prevent it from replacing other imaging modalities.

## Conclusion

An increase of more than 6 mm in the anteroposterior diameter of the renal pelvis after furosemide injection could serve as a diagnostic criterion in MRU for pediatric ureteropelvic junction obstruction, with high sensitivity and specificity. There is no significant difference in the increase in renal pelvis diameter after furosemide injection according to the etiology of ureteropelvic junction obstruction (intrinsic vs. extrinsic). MRI can reliably detect the presence of a crossing vessel but cannot confirm whether it is the cause of ureteropelvic junction obstruction. Certain factors could predict the etiological role of a crossing vessel in ureteropelvic junction obstruction, but these factors remain unknown. MRI is a promising imaging modality which, with technological advances – in particular the development of non-contrast angiography and diffusion sequences, and the broader use of functional studies – should play an increasingly important role in the investigation of ureteropelvic junction obstruction.

## Data Availability

No datasets were generated or analysed during the current study.

## References

[1] European Association of Urology (2023) EAU Guidelines 2023. Presented at the EAU Annual Congress, Milan

[2] Williams B, Tareen B, Resnick MI (2007) Pathophysiology and treatment of ureteropelvic junction obstruction. Curr Urol Rep 8:111–117 17303015 10.1007/s11934-007-0059-8

[3] European Society for Paediatric Urology. Paediatric Urology Web-Book. 2015. Available from: https://www.espu.org/e-books/ESPU_Paediatric_Urology_book/. Accessed July 2025

[4] Olsen LH, Rawashdeh YFH (2016) Surgery of the ureter in children. In: Wein AJ, Kavoussi LR (eds) Campbell-Walsh Urology, 11th edn. Elsevier, Philadelphia, pp 3057–3066

[5] Houat AP, Guimarães CTS, Takahashi MS et al (2021) Congenital anomalies of the upper urinary tract: a comprehensive review. Radiographics 41:462–486 33513074 10.1148/rg.2021200078

[6] Hashim H, Woodhouse CRJ (2012) Ureteropelvic junction obstruction Eur Urol Suppl 11:25–32

[7] Press B, Cho J, Kirsch AJ (2025) Magnetic resonance urogram in pediatric urology: a comprehensive review of applications and advances. Int Braz J Urol 51:e20250047 40079923 10.1590/S1677-5538.IBJU.2025.0047 PMC12052027

[8] Grattan-Smith JD, Little SB, Jones RA (2008) Mr urography evaluation of obstructive uropathy. Pediatr Radiol 38:49–69 10.1007/s00247-007-0667-y 18071689

[9] Vivier PH, Blondiaux E, Dolores M et al (2009) Uro-IRM fonctionnelle chez l’enfant. J Radiol 90:11–19 19182709 10.1016/s0221-0363(09)70073-3

[10] Rosi P, Virgili G, Stasi SMD et al (1990) Diuretic ultrasound: a non-invasive technique for the assessment of upper tract obstruction. Br J Urol 65:566–569 2196970 10.1111/j.1464-410x.1990.tb14821.x

[11] Goldberg SD, Witchell S, Drohomyrecky A, Louis ESt (1988) Diuretic ultrasound: technique for assessment of obstructed renal unit. Urology 32:546–548 3059661 10.1016/s0090-4295(98)90042-5

[12] Sarafidis PA, Georgianos PI, Lasaridis AN (2010) Diuretics in clinical practice. Part I: mechanisms of action, pharmacological effects and clinical indications of diuretic compounds. Expert Opin Drug Saf 9:243–257 20095917 10.1517/14740330903499240

[13] Ucar AK, Kurugoglu S (2020) Urinary ultrasound and other imaging for ureteropelvic junction type hydronephrosis (UPJHN). Front Pediatr 8:546 33042907 10.3389/fped.2020.00546 PMC7526330

[14] Shoma AM, El Nahas AR, Bazeed MA (2007) Laparoscopic pyeloplasty: a prospective randomized comparison between the transperitoneal approach and retroperitoneoscopy. J Urol 178:2020–2024 17869300 10.1016/j.juro.2007.07.025

[15] Parikh KR, Hammer MR, Kraft KH et al (2015) Pediatric ureteropelvic junction obstruction: can magnetic resonance urography identify crossing vessels? Pediatr Radiol 45:1788–1795 26216155 10.1007/s00247-015-3412-y

[16] Pavicevic PK, Saranovic DZ, Mandic MJ et al (2015) Efficacy of magnetic resonance urography in detecting crossing renal vessels in children with ureteropelvic junction obstruction. Ann Ital Chir 86:443–449 (PMID: 26567456) 26567456

[17] Perez-Brayfield MR, Kirsch AJ, Jones RA, Grattan-Smith JD (2003) A prospective study comparing ultrasound, nuclear scintigraphy and dynamic contrast-enhanced magnetic resonance imaging in the evaluation of hydronephrosis. J Urol 170:1330–1334 14501762 10.1097/01.ju.0000086775.66329.00

[18] Damasio MB, Sertorio F, Wong MCY et al (2022) Functional magnetic resonance urography in ureteropelvic junction obstruction: proposal for a pediatric quantitative score. Front Pediatr 10:882892 35783310 10.3389/fped.2022.882892 PMC9243529

